# Fat Replacers in Baked Food Products

**DOI:** 10.3390/foods7120192

**Published:** 2018-11-25

**Authors:** Kathryn Colla, Andrew Costanzo, Shirani Gamlath

**Affiliations:** Centre for Advanced Sensory Sciences, School of Exercise and Nutrition Sciences, Deakin University, 1 Gheringhap Street, Geelong 3220, Australia; k.colla@deakin.edu.au (K.C.); andrew.costanzo@deakin.edu.au (A.C.)

**Keywords:** fat replacers, baked products, carbohydrates, gums, gels, whole foods

## Abstract

Fat provides important sensory properties to baked food products, such as colour, taste, texture and odour, all of which contribute to overall consumer acceptance. Baked food products, such as crackers, cakes and biscuits, typically contain high amounts of fat. However, there is increasing demand for healthy snack foods with reduced fat content. In order to maintain consumer acceptance whilst simultaneously reducing the total fat content, fat replacers have been employed. There are a number of fat replacers that have been investigated in baked food products, ranging from complex carbohydrates, gums and gels, whole food matrices, and combinations thereof. Fat replacers each have different properties that affect the quality of a food product. In this review, we summarise the literature on the effect of fat replacers on the quality of baked food products. The ideal fat replacers for different types of low-fat baked products were a combination of polydextrose and guar gum in biscuits at 70% fat replacement (FR), oleogels in cake at 100% FR, and inulin in crackers at 75% FR. The use of oatrim (100% FR), bean puree (75% FR) or green pea puree (75% FR) as fat replacers in biscuits were equally successful.

## 1. Introduction

Dietary fat has an important role within food matrices beyond basic nutrition. It contributes to many sensory and quality properties of a food including physical, textural and olfactory factors which all influence overall palatability. Many snack foods, in particular, rely on dietary fat to fulfil these palatable qualities in order to maintain consumer acceptance and consumption. The World Health Organisation [1], along with many national health authorities [2,3,4,5], recommends decreasing consumption of discretionary snack foods due to their poor nutritional content. Excess dietary fat intake, notably from discretionary snack foods, is one of the key contributors to excess energy intake and therefore weight gain [6]. Prevalence of overweight and obesity is rising worldwide [7,8] which is cause for concern as obesity is associated with increased risk of cardiovascular disease [9], type 2 diabetes mellitus [10], and some cancers [11].

Despite consumer awareness and product labelling [12,13], consumption of snack foods is relatively high with little compensation for the increased energy intake [14,15,16]. Many promoters have been attributed to increased snack food intake, such as convenience, taste, marketing and pricing [16,17]. In order to respond to these recommendations and consumer demands, manufacturing companies are increasingly developing snacks which are more nutrient dense than traditional snacks such as chips and cakes, which are typically high in added fat, sugar and sodium. Some examples of these types of innovative snacks include yoghurts, bars, puddings, crackers and chips which contain popular health foods (or superfoods) such as seeds, nuts, ancient grains, other wholegrains, dietary fibres, legumes, fruits and vegetables. While many of these snacks may be high in protein and dietary fibre, many also typically contribute large amounts of fat, sugar and sodium to the consumers’ diet [18]. Efforts must be made to develop appealing snacks which are both high in protein and dietary fibre while not contributing large amounts of sodium, sugar and fat. Snack food categories such as cakes and muffins are yet to see significant innovation in creating high protein or high fibre alternatives [16]. In addition, there are still limited reduced fat options of these baked products on the market, likely attributed to the technological difficulty in producing such products. Ultimately, there is a need to increase the number of nutritious snack options available that satisfy the above drivers, while reducing fat composition and therefore total energy intake. Baked snack foods that omit dietary fat as a “low-fat” alternative often have poor sensory properties, such as crumbliness, dryness, poor mouthfeel and overall reduced consumer acceptance [19,20,21,22,23]. A number of potential “fat replacers” have been purported in order to reduce the fat content in food matrices whilst maintaining the sensory properties that are usually attributed to dietary fat. Fat replacers are subcategorised as either fat substitutes or fat mimetics. Fat substitutes replicate the functional and sensory properties of fat in a food, usually contain no energy or less energy than fat, and may be used to replace some or all of the fat normally present in a product [24,25]. Fat mimetics are protein- or carbohydrate-based ingredients that are not used to fully substitute the use of fat, but rather replicate some of the properties that fat provides within a food [24,25]. Many baked products on the market currently utilise fat replacers in order to reduce the total energy or fat content whilst maintaining consumer acceptance. This review aims to summarise the current evidence for application of fat replacers in biscuits, crackers, muffins, cakes and bread, and their effect on quality and sensory properties.

## 2. Application of Fat Replacers in Baked Products

Fat replacers are defined by the American Dietetic Association as “an ingredient that can be used to provide some or all of the functions of fat, yielding fewer calories than fat” [24]. A wide range of products in the food industry uses fat replacers, some of which include meat, dairy and baked products [24]. It is important for product developers and food technologists to understand how different fat replacers influence the sensory and physical quality of snacks in order to guide the development of healthier alternative products. For example, in cakes, fat can contribute to increased leavening, tenderness and a finer crumb through a combined effect of trapping air cells during the creaming process [26]. This structure is then set during baking due to starch gelatinisation and coagulation of egg proteins [26]. Fat is typically used in biscuits to lubricate and coat the flour granules to prevent water absorption, and the development of starch and gluten in order to achieve a fine crumb (crumbly texture) and soft, tender mouthfeel [27]. Fat also contributes other important functions to cakes, biscuits and crackers such as flavour delivery and shelf life which is achieved through delaying water absorption by starch granules [28,29,30,31].

Fat replacers can be ingredients which are of carbohydrate, protein or fat origin, with many different types of fat replacers with different structures and functions within each group. We have not differentiated fat substitutes and fat mimetics in this review as the majority of fat replacers used in baked food products are fat mimetics. Instead, we have categorised fat replacers in this review as complex carbohydrate powders, gums and gels, whole food purees and products, or a combination thereof. This categorisation is based on their functional and industrial applications rather than their chemical properties.

(a) Complex carbohydrates are typically successful fat replacers due to their ability to bind water to form a paste which can mimic the texture and viscosity of fats in food products through providing lubricant or flow properties similar to fat in some food systems [28,32]. Examples of carbohydrate based fat replacers include inulin, maltodextrin and plant fibres.
(b) Gums and gels work similarly in function to complex carbohydrates, in that they bind with water to form gels which mimic the texture and viscosity of fats [28]. While some gums and gels are made up of complex carbohydrates, this is not specific as there are some protein- and fat-based gums and gels. Examples of gums and gels used as fat replacers include pectins, oleogels and whey protein.
(c) Whole foods are complete or partial food matrices that are included in a food product as fat replacers. Recently, many products have utilised whole foods such as fruits and vegetables, legumes or cereal based ingredients as fat replacers. These foods are typically successful due to their highly creamy texture when mashed or processed. Foods such as avocado can achieve this due to its oil composition, banana for its high starch content, and legumes for their high starch and protein contents.
(d) Combinations of the above fat replacers are useful as they can potentially replicate multiple sensory qualities of dietary fat. In addition, complexes formed from these combinations, such as emulsions and esters, may have a greater fat replacing effect than the sum of their parts.

## 3. Summary of the Current Fat Replacers Used in Baked Products

Complex carbohydrate fat replacers range from digestible starches to non-digestible plant fibres (Table 1). It should be noted that the replacement of dietary fat with complex carbohydrates reduced energy density of all the food products in Table 1, regardless of fibre status, due to complex carbohydrate being less energy dense than fat. The use of fibres instead of starches could have an advantage on the market, as foods may meet criteria for fibre content claims. Inulin, a non-digestible dietary fibre typically derived from chicory root, was observed to have the greatest success in replacing dietary fat in baked products, where a fat replacement (FR) level of up to 75% in legume crackers and cake (1:1, inulin: water; and 1:2 inulin: water, respectively) was able to reduce total energy without any changes in consumer acceptance [33,34]. It should be noted that the addition of inulin did change the textural and physical properties of the cracker and cake products. While acceptance was not measured for the use of inulin in muffins, 50% FR had the least sensory and physical changes compared to 75% and 100% FR [35]. In addition, Zahn et al. tested the use of four commercial inulin formulations in muffins which varied in inulin to water ratio and solubility, but the outcomes for each were similar [35]. Maltodextrin was also successful at 75% fat replacer level in legume crackers and at 66% FR in muffins, although there were changes noted in aroma, appearance, taste and texture [33,36]. Total FR of inulin or maltodextrin (100%) had a significant decrease in consumer acceptance, so it is not recommended to fully replace fat in a baked food product. Results were also promising for inulin used as a fat replacer in biscuits, although there was some notable changes to textural and physical properties [33,34,35,37,38,39]. Other complex carbohydrates used as fat replacers in biscuits included lupine extract, maltodextrin, corn fibre, and rice starch, although all of these had significant effects on sensory properties of the biscuits except rice starch [33,36,40,41,42,43]. Rice starch has no significant effects on sensory properties, but was only tested at 20% FR. All complex carbohydrate fat replacers had a significant effect on the physical properties of doughs and their baked products, with significant increases in density, toughness, breaking strength, moisture, and decreases in volume for nearly all tested products [33,34,35,36,37,38,39,40,41,42,43,44,45,46]. 

Of all the complex carbohydrate fat replacers, inulin had the greatest success at reducing total fat and energy of the food product, with the least impact on sensory qualities and consumer acceptance, particularly in the legume crackers. Long chain inulin has the ability to form microcrystals which in turn aggregate together, interact with water, and eventually agglomerate creating a gel network [47]. To some extent, this gel network seems to have the ability to mimic the functions of fat in baked products such as being able to lubricate dry ingredients (through surrounding starch and protein), assisting in maintaining a shortening effect. Maltodextrin was also a successful fat replacer in legume crackers, although it was not as successful at replacing fat in biscuits compared to inulin. Inulin is also a good source of fibre, has promising gut health properties due to its prebiotic nature, and may increase absorption of nutrients such as calcium [48]. Moreover, inulin may benefit from marketing with fibre content claims, which may be appealing to consumers. Therefore, we recommend inulin as a reasonably high level fat replacer in crackers, cakes, biscuits and muffins [48]. 

Gum and gel fat replacers, while mostly being carbohydrates, also include lipid-based and protein-based gums and gels (Table 2). Some of these fat replacers may also increase suitability for nutrition content claims, such as sources of fibre or protein. Guar gum and xanthan gum had relatively little effect on the physical properties of the cake product when used as fat replacers [49]. While sensory measures were not compared to a control, both types of cakes were rated as acceptable with a greater acceptance in the cake containing xanthan gum, and 50% FR was considered ideal [49]. Oatrim (a tasteless white powder derived from oats, comprised of amylodextrins and 5–10% β-glucan soluble fiber; incorporated as a powder or gel), caused significant changes to the physical properties of cake, croissants and biscuits [50,51,52]. However, this did not appear to have any impact on the sensory properties of these foods, even at 100% FR. Pectin also caused significant changes to the physical properties of cake, croissants and biscuits, specifically increasing the hardness and reducing the volume of these foods, which was paralleled by increased perception of hardness and reduced flavour from sensory evaluations [42,46,53,54]. This is notable as pectin was tested at a relatively low FR level in cake and biscuits (10–30%), suggesting it is not an ideal fat replacer in baked products. Hydroxypropyl methylcellulose (HPMC) had significant effects on physical and sensory properties of crackers and biscuits, even at relatively low FR levels [33,55]. While consumer acceptance was not tested on crackers due to being considered unacceptable by a focus group [33], HPMC in biscuits was considered significantly less acceptable compared to control biscuits containing 18% canola oil suggesting it is also not an ideal fat replacer in these foods. 

Oleogels are products of solidifying vegetable oils using natural wax esters [56,57,58,59]. The oleogelation process forms waxy crystal structure which hold liquid oil within a solid matrix, which allows the use of liquid vegetable oils in place of shortening. While this does not necessarily reduce the total fat content of a food product, it is useful in reducing saturated fat content. It should be noted that all oleogels studies reviewed in this paper did reduce overall fat content of their tested foods [56,57,58,59]. However, oleogels were not successful as fat replacers in these studies as they made biscuit and cake denser and harder. Sensory properties seemed to be promising with an increase in taste and no difference in acceptance compared to the control foods. Lastly, whey protein was also not an ideal fat replacer for biscuits as it resulted in a decrease in overall flavour and acceptance [45,46]. 

Overall, gums and gels were not very successful as fat replacers in baked goods. Oatrim appeared to be the most successful as there were no significant changes to the sensory properties of cake and biscuits, although there were a large range of physical changes to these foods which might have an impact on industrial applications. Xanthan gum and guar gum might potential be useful fat replacers in cake as they had little impact on physical properties, although more robust sensory evaluations are needed in future studies. 

The interest in using whole food fat replacers has increased in recent years. These fat replacers are beneficial as they have a range of carbohydrates, lipids and proteins that may aid in the rheological properties of baked products, making them potentially more suitable than simple extracts and isolates. Overall, whole food fat replacers had the least effect on the physical and sensory properties of baked products, and in some cases increased the consumer acceptance (Table 3). Apricot kernel flour was a successful fat replacer with little impact on the physical and sensory properties of biscuits at a maximum of 50% FR [60,61]. Chia seed mucilage also had little impact on physical properties of cake and bread up to 100% FR [62,63], although sensory properties were not tested in these studies. High oleic sunflower oil (HOSO) did not significantly decrease the amount of total fat in biscuits, but did reduce the saturated fat content [64]. However, the use of HOSO as a fat replacer was not considered successful as it has significant impact on the volume, colour and texture of the biscuits. The use of avocado puree as a fat replacer in cake and biscuits was successful at 50% FR, as it did not impact consumer acceptance [51,65]. However, at 75–100% FR, acceptance of the low-fat cake decreased compared to the control cake containing shortening [65]. Apple puree or pomace was the only whole food fat replacer to result in a reduction in sensory quality and consumer acceptance, even at low FR levels (10%) [66,67]. Therefore, apple puree is not recommended as a fat replacer in biscuits. Bean puree and green pea puree had very similar effects on the sensory properties of biscuits with increases in sensory qualities at 25–75% FR [68,69]. The use of green pea puree at FR of 25% in biscuits was considered ideal, whereas a FR of 100% resulted in reduced consumer acceptance [69]. Lastly, a high β-glucan product derived from oats or oat bran had significant impact on texture, colour and moisture of biscuits [45,54,70]. Although sensory properties were not tested in these studies, this suggests that the high β-glucan product was not a successful replacer for shortening in biscuits. 

Whole foods may be the most suitable candidates for fat replacers in baked foods as they appeared to have the least impact on physical and sensory properties. In addition, they may also be beneficial as they may contain phytochemicals and micronutrients which could increase the health benefits and marketing potential of baked foods products, leading to novel functional foods. Lastly, consumer are more likely to accept foods with ingredients or additives that are made from natural, whole food products [71]. Bean and pea purees were the most successful fat replacers for biscuits at 25–75% FR, and avocado puree was successful at reducing fat in cake at 50% FR. However, more studies on whole food fat replacers in biscuits and bread is needed before they can be recommended as reliable fat replacers. 

Fat replacers in combination with additional ingredients may provide better fat-like qualities as the additional ingredients are usually designed to supplement the unwanted effects of individual fat replacers, as seen above (Table 1, Table 2 and Table 3). These additional ingredients are usually other types of fat replacers, but can also be enzymes or emulsifiers. Few studies have assessed combined fat replacers in baked products, although the results appear promising (Table 4). Polydextrose and guar gum were successful fat replacers in biscuits at a relatively high level of FR (70%), with an increase in perceived taste, flavour and consumer acceptance [72]. Maltodextrin and xanthan gum yielded increased moisture, hardness and chewiness in 66% FR muffins, but sensory analysis was not conducted in these samples [36]. Kel-Lite BK, a commercial fat replacer containing xanthan gum, guar gum, cellulose gel, sodium stearoyl lactylate, gum Arabic, dextrin, lecithin, and mono- and diglyceride, resulted in increased bitterness and, oddly increased both crumb firmness and softness in biscuits at 33%, 66% and 100% FR [54]. HOSO and inulin were also successful fat replacers in biscuits at 100% FR [64,73], although HOSO does contain lipids so the biscuits only had reduced saturated fat rather than total fat. However, HOSO and inulin resulted in decreased appearance, flavour, odour, texture, and consumer acceptance in cakes, croissants and muffins [73]. Therefore, HOSO and inulin may only be suitable for use as fat replacers in biscuits. HOSO and β-Glucan may also be a useful fat replacer at 100% FR as this had little impact on physical properties in biscuits, although sensory evaluations were not conducted [64]. A combination of emulsion filled gel based on inulin and extra virgin olive oil (EVOO) has also been trialed as a fat mimetic in biscuits [74]. At 50% FR, there were no changes to the physical properties and the overall consumer acceptance of the biscuit compared to the control biscuit containing 20% butter, although there was a decrease in overall flavour. However, consumer acceptance was not maintained at 100% FR. Inulin, lipase and a commercial emulsifier (“Colco”; a type of alpha-gel emulsifier containing glycerol monostearate and polyglycerol esters of fatty acids) had little impact on physical properties of cake at 50–70% FR, although no sensory evaluation was conducted for this combined fat replacer either [75]. One study assessed the double, but not triple, combinations of corn fibre, maltodextrin and lupine extract in biscuits, each at 30–40% FR [40]. All combinations had little impact on the physical properties of the biscuits compared to the control biscuit containing 33% margarine. However, consumer preference for corn fibre and lupine extract was significant lower than the control, whereas corn fibre and maltodextrin was significant higher than the control [40]. This suggests that the combination of corn fibre and maltodextrin may be an ideal fat replacement in biscuits at a moderate FR level. Tapioca dextrin, tapioca starch and resistant starch as a combination fat replacer had an impact on a wide range of sensory properties in biscuits [76]. However, overall consumer acceptance decreased, even at relatively low FR levels (10–20%), so we do not recommend the use of this combination fat replacer in biscuits. 

Overall, combination fat replacers may be potential candidates for ingredients in low-fat baked products. The use of polydextrose and guar gum appears to be a reasonably effective fat replacer in biscuits. However, with the limited evidence currently available, recommendations cannot be made for the use of combination fat replacers in other baked products. 

## 4. Industry Recommendations and Conclusions

It should be noted that there is limited literature on the use of fat replacers in low-fat baked products. Many of the reviewed fat replacers have only been assessed once, and also only in one type of food. There is a need for additional replicate studies using a variety of recipes. Also, while we have reviewed the current literature here, we cannot compare physical and sensory properties between studies. Therefore, while we can summarise which fat replacers were successful within a certain baked product, it is difficult to determine which fat replacer is best. In addition, the use of fat replacers in bread, muffins and croissants were only assessed in few studies each. Therefore, there is not enough information to make a recommendation of the best type of fat replacer for these products. Below is our recommendations for the best currently assessed fat replacers in a range of baked food products:

**Biscuit**—Oatrim was the most successful fat replacer in biscuits as it was able to retain most sensory properties of a traditional biscuit even at 100% FR, although there was a decrease in hardness and brittleness [51,52]. However, it should also be noted that both bean puree and green pea puree were able to increase the sensory qualities and consumer acceptance of biscuits at 75% FR with less of an impact on the physical properties compared to oatrim [68,69]. Legume purees might also have an advantage over oatrim as they may aid the marketability of food products due to potential nutrition claims such as vegetable and protein content. However, legume purees should not be used at 100% FR. Overall, we recommend the use of either oatrim or legume purees as fat replacers in biscuits. 

**Cake**—Oleogels appeared to be the most successful fat replacer in cake, with no changes to the sensory qualities at 100% FR [57,58,59]. However, there were significant changes to the physical properties of cake when using oleogels at FR levels ≥50% [58] which might lead to difficulty during cake production. An alternative could be avocado puree which was only successful at 50% FR but had less of an impact on the physical properties of cake [65], or inulin which was successful up to 75% FR but had an impact on the physical and textural properties of cake [34]. 

**Cracker**—While there was only one study on the use of fat replacers in crackers [33], it assessed and compared a range of fat replacers in the one study. Inulin appeared to be the most successful fat replacer in these crackers, reaching an acceptable level of FR at 75%. The additional benefits of using inulin is that it may aid the marketability of food products due to potential high fibre claims. 

## Figures and Tables

**Table 1:** Summary of quality changes of complex carbohydrate fat replacers in baked food products.

Fat Replacer	Food(s)	FR Tested	Quality Changes
Inulin	Legume Cracker [33]	25–100%	Physical: ↓ cell density, aW, volume; ↑ breaking strength, dough consistency, moisture, crumb density, firmness, springiness; NSC cohesiveness Sensory: ↓ buttery flavour, crumbliness, acceptance (100% FR), embrowning (muffin: 50%), open surface, arched shape, typical smell, sweetness, typical taste; ↑ toasted flavour, chewiness, adhesiveness, springiness (100% FR), crispiness, crunchiness, dryness, toughness hardness, glossiness; NSC acceptance (50–75% FR)
	Cake [34]	9.3–50%	
	Biscuit [37,38,39]	35–100%	
	Muffin [35]	50–100%	
Lupine Extract	Biscuit [40]	30–40%	Physical: ↓ volume, lightness; ↑ breaking strength, dough consistency, aW, moisture Sensory: ↓ sweetness; ↑ firmness, dryness, chewing time, roasted flavour
Maltodextrin	Biscuit [40,41]	30–40%	Physical: ↓ volume, spread ratio; ↑ breaking strength, dough consistency, aW, moisture, cohesiveness, chewiness; NSC hardness, springiness Sensory: ↓ sweetness, overall flavour, aroma, colour, appearance, texture, taste, flavor, overall acceptance (muffin); ↑ firmness, dryness, chewing time; NSC acceptance (legumes cracker: 50–75% FR), mouthfeel
	Legume Cracker [33]	25–100%	
	Muffin [36]	66%	
	Croissant [42]	25–100%	
Corn Fibre	Biscuit [40]	30–40%	Physical: ↓ volume; ↑ breaking strength, dough consistency, aW, moisture Sensory: ↓ sweetness; ↑ firmness, dryness, chewing time
Rice Starch	Biscuit [43]	20%	Physical: ↓ volume, height (muffins); ↑ thickness (biscuit) Sensory: NSC all sensory qualities
	Muffin [43]	20%	
Resistant Starch	Biscuit [34]	40%	Physical: ↓ hardness; ↑ spread ratio
Polydextrose	Biscuit [41,45,46]	11.5–50%	Physical: ↑ hardness, brittleness, aW, breaking strength; ↓ penetration distance, spread ratio [41]
			Sensory: ↑ hardness, ↓ overall flavour, appearance, texture, taste, acceptance, NSC colour

aW: Water activity; FR: Fat replacement; NSC: No significant change; ↓: decrease; ↑: increase.

**Table 2:** Summary of quality changes of gum and gel fat replacers in baked food products.

Fat Replacer	Food(s)	FR Tested	Quality Changes
Xanthan Gum	Cake [49]	25–100%	Physical: ↓ volume (100% FR), elasticity; ↑ dough density; NSC aW, firmness
Guar Gum	Cake [49]	25–100%	Physical: ↓ volume (100% FR), elasticity; ↑ dough density; NSC aW, firmness
Oatrim	Cake [50]	20–60%	Physical: ↓ air bubbles, viscosity, spread ratio, moisture, hardness (biscuits), brittleness; ↑ specific gravity, dough pH, height, hardness (cake), cohesiveness, springiness, aW Sensory: NSC colour, appearance, tenderness, sweetness, flavour, aftertaste, overall
	Biscuit [51,52]	50–100%	
Pectin	Cake [53]	10–30%	Physical: ↓ spread ratio, penetration distance, volume; ↑ weight, aW, breaking strength, specific gravity, moisture, hardness Sensory: ↑ hardness, lightness, bitterness (biscuit: 100%); ↓ overall flavour, acceptance, colour, texture, cell size, taste, mouthfeel
	Biscuit [46,54]	10–100%	
	Croissant [42]	25–100%	
Hydroxypropyl Methylcellulose (HPMC)	Legume Cracker [33]	25–100%	Physical: ↓ lightness, yellowness; ↑ moisture, hardness, breaking strength; NSC aW Sensory: ↑ hardness, crispness; ↓ overall acceptance, yellowness, buttery flavour
	Biscuit [55]	15–30%	
Oleogels	Biscuit [56]	40–70%	Physical: ↓ spread ratio, breaking strength, specific volume, fragmentation, porosity; ↑ hardness, specific gravity; NSC cell structure Sensory: ↑ hardness, chewiness, springiness, lightness (crust), colour (crust), overall taste; ↓ cohesiveness; NSC overall smell, overall acceptability
	Cake [57,58,59]	25–100%	
Whey Protein	Biscuit [45,46]	11.5–50%	Physical: ↓ hardness, weight; ↑ aW; NSC spread ratio Sensory: ↑ hardness; ↓ overall flavour, acceptance

aW: Water activity; FR: Fat reduction; NSC: No significant change; ↓: decrease; ↑: increase.

**Table 3:** Summary of quality changes of whole food fat replacers in baked food products.

Fat Replacer	Food(s)	FR Tested	Quality Changes
Apricot Kernel Flour	Biscuit [60,61]	10–50%	Physical: ↓ spread ratio, yellowness; ↑ hardness, lightnessSensory: NSC overall sensory score
Chia Seed Mucilage	Cake [62,63]	25–100%	Physical: ↓ lightness, yellowness; ↑ firmness; NSC volume, symmetry, uniformity, redness, moisture, aW, breaking strength
	Bread [63]	25–100%	
High Oleic Sunflower Oil (HOSO)	Biscuit [64]	100%	Physical: ↓ volume, moisture, lightness, yellowness; ↑ biscuit density, breaking strength, redness; NSC dough density
Avocado Puree	Biscuit [51]	50%	Physical: ↓ spread ratio, moisture, stiffness, hardness; ↑ aW, brittleness Sensory: ↓ appearance, acceptance (75–100%); NSC colour, tenderness, sweetness, flavour, aftertaste, acceptance (50%), overall sensory score
	Cake [65]	50–100%	
Apple Puree/Pomace	Biscuit [66,67]	10–100%	Physical: ↓ spread ratio, brittleness, hardness, yellowness; ↑ moisture Sensory: ↓ appearance, texture, chewiness, sweetness, moistness (100%), flavour, aftertaste, overall sensory score; ↑ moistness (50%); NSC colour
Bean Puree	Biscuit [68]	25–75%	Sensory: ↑ appearance, colour, flavour, texture, acceptance
Green Pea Puree	Biscuit [69]	25–100%	Physical: ↑ moisture Sensory: ↓ flavour, aftertaste, acceptance (100%); ↑ colour, moistness, flavour (25–75%), acceptance (25%); NSC smell
Oat Bran/High β-Glucan Oat Product	Biscuit [45,54,70]	10–100%	Physical: ↓ spread ratio, hardness [59], redness, yellowness; ↑ hardness [37], brittleness, moisture, aW, lightness, volume

aW: Water activity; FR: Fat reduction; NSC: No significant change; ↓: decrease; ↑: increase.

**Table 4:** Summary of quality changes of combined fat replacers in baked food products.

Fat Replacer	Food(s)	FR Tested	Quality Changes
Polydextrose and Guar Gum	Biscuit [72]	70%	Physical: ↑ spread ratio, hardness, stress-strain ratio, moisture Sensory: ↑ overall taste, overall flavour, acceptance
Maltodextrin and Xanthan Gum	Muffin [36]	66%	Physical: ↑ aW, moisture, hardness, chewiness; ↓ volume; NSC springiness, cohesiveness
Kel-Lite BK	Biscuit [54]	33–100%	Physical: ↑ crumb firmness, crumb softness; NSC volume Sensory: ↑ bitterness
HOSO and Inulin	Biscuit [64,73]	100%	Physical: NSC dough density, biscuit density, volume, moisture, breaking strength, lightness, colour Sensory: ↓ appearance (croissant and muffin), odour (croissant and muffin), texture (cake and croissant), flavour (cake and muffin), acceptance (cake, croissant and muffin), purchase intent (cake), preference (cake and muffin)
	Cake [73]		
	Croissant [73]		
	Muffin [73]		
HOSO and β-Glucan	Biscuit [64]	100%	Physical: ↓ volume, lightness; ↑ biscuit density; NSC dough density, moisture, breaking strength, colour
EVOO and EFG based on Inulin	Biscuit [74]	50–100%	Physical: ↓ breaking strength (100%), porosity (100%); Sensory: ↓ caramel odour, buttery odour and flavour, sweetness, crunchiness, crush, dryness, acceptance (100%); ↑ consistency; NSC grain odour and flavour, saltiness
Inulin, Lipase and Emulsifier	Cake [75]	50–70%	Physical: ↓ batter density; ↑ cohesiveness; NSC volume, cell structure, hardness, chewiness, springiness
Corn Fibre, Maltodextrin and/or Lupine Extract	Biscuit [40]	30–40%	Physical: ↓ lightness, volume; ↑ breaking strength Sensory: ↓ preference (corn fibre and lupine extract); ↑ preference (corn fibre and maltodextrin)
Tapioca Dextrin, Tapioca Starch and Resistant Starch	Biscuit [76]	10–20%	Physical: ↓ spread ratio; ↑ breaking strength Sensory: ↓ buttery taste, crunchiness, hardness, colour, buttery odour, appearance, texture, taste, sweetness, acceptance; ↑ shape homogeneity, floury taste, pastiness, floury odour

aW: Water activity; FR: Fat reduction; NSC: No significant change; ↓: decrease; ↑: increase, HOSO: High Oleic Sunflower Oil; EVOO: Extra Virgin Olive Oil; EFG: Emulsion Filled Gel.
